# Risk factors for acute respiratory distress syndrome in sepsis patients: a retrospective study from a tertiary hospital in China

**DOI:** 10.1186/s12890-022-02015-w

**Published:** 2022-06-21

**Authors:** Yuequan Shi, Liang Wang, Sihan Yu, Xiaochun Ma, Xu Li

**Affiliations:** 1 Department of Respiratory and Critical Care Medicine, Peking Union Medical College Hospital, Chinese Academy of Medical Sciences & Peking Union Medical College, Beijing, China; 2 Department of Critical Care Medicine, The First Affiliated Hospital of China Medical University, Shenyang, China

**Keywords:** Acute respiratory distress syndrome, Sepsis, Risk factor, Severity

## Abstract

**Background:**

Less is known about the risk factors for acute respiratory distress syndrome (ARDS) in sepsis patients diagnosed according to sepsis 3.0 criteria. Moreover, the risk factors for ARDS severity remain unclear.

**Methods:**

We retrospectively collected the characteristics of sepsis patients from the intensive care unit of the First Affiliated Hospital of China Medical University from January 2017 to September 2018. Logistic regression was used in determining the risk factors.

**Results:**

529 patients with sepsis were enrolled and 179 developed ARDS. The most common infection sites were acute abdominal infection (n = 304) and pneumonia (n = 117). Multivariate analysis showed that patients with pancreatitis with local infection (odds ratio [OR], 3.601; 95% confidence interval [CI], 1.429–9.073, *P* = 0.007), pneumonia (OR 3.486; 95% CI 1.890–6.430, *P* < 0.001), septic shock (OR 2.163; 95% CI 1.429–3.275, *P* < 0.001), a higher sequential organ failure assessment (SOFA) score (OR 1.241; 95% CI 1.155–1.333, *P* < 0.001) and non-pulmonary SOFA score (OR 2.849; 95% CI 2.113–3.841, *P* < 0.001) were independent risk factors for ARDS. Moreover, pneumonia is associated with increased severity of ARDS (OR 2.512; 95% CI 1.039–6.067, *P* = 0.041).

**Conclusions:**

We determined five risk factors for ARDS in sepsis patients. Moreover, pneumonia is significantly associated with an increased severity of ARDS.

## Introduction

Acute respiratory distress syndrome (ARDS) remains a major clinical syndrome in the intensive care unit (ICU). It had been reported that 10.4% of total ICU-admitted patients developed ARDS. Moreover, nearly 40% of ARDS patients were underdiagnosed [1]. Sepsis, defined as life-threatening organ dysfunction caused by a dysregulated host response to either pneumonia or non-pulmonary infection, is a common risk factor for ARDS. Patients with sepsis-induced ARDS were reported to have a poorer prognosis than those without ARDS [2, 3] due to the lack of effective treatment strategies [4–8]. In accordance with the rapid deterioration of such clinical conditions, clinicians have shifted the management paradigm to prevent the development of ARDS [9]. Potential risk factors for ARDS have been reported in previous studies [10–14], but there is still a lack of large-sample studies on risk factors for ARDS in ICU sepsis patients diagnosed by updated sepsis 3.0 criteria [15]. Moreover, to our knowledge, the risk factors related to the severity of ARDS in ICU sepsis patients diagnosed according to sepsis 3.0 criteria have not been reported to date.

In this study, we evaluated risk factors for ARDS and those associated with ARDS severity in sepsis patients in ICU diagnosed according to sepsis 3.0 criteria.

## Methods

### Study population

We retrospectively collected the clinical records of adult (≥ 18 years old) patients with sepsis who were admitted to the ICU of the First Affiliated Hospital of China Medical University from January 1st, 2017 to September 30th, 2018. Sepsis was diagnosed in accordance with the Third International Consensus Definitions for Sepsis and Septic Shock [15]. Exclusion criteria were as follows: (1) patients who underwent cardiopulmonary resuscitation (CPR); (2) patients with advanced solid or hematological tumors; (3) patients currently diagnosed with cirrhosis; (4) patients who underwent organ transplantation; and (5) patients with regular administration of hormones or immunosuppressors.

### Data collection and outcomes

Clinical records were collected regarding demographic characteristics, including age, sex, underlying disease, smoking and drinking history, and site of infection of each patient. The baseline acute physiology and chronic health evaluation II (APACHE II) score, sequential organ failure assessment (SOFA) score, SOFA score excluding respiratory function (non-pulmonary SOFA score), and presence of septic shock before the development of ARDS were used to assess the severity of disease. We also included baseline laboratory data, such as PH, lactate (Lac), serum creatinine (Scr)and platelet value (PLT). The mechanical ventilation hours, length of ICU stay, length of hospital stay, and ICU mortality were recorded in detail. The 28-day mortality and 90-day mortality were assessed during follow-up. Sepsis patients were divided into 2 groups based on whether they developed ARDS. ARDS was diagnosed according to Berlin definition [16]. In terms of patients with ARDS, we further classified them into 3 groups based on the severity according to Berlin definition which is based on the oxygenation index (mild: 200 mmHg < PaO_2_/FiO_2_ ≤ 300 mmHg, moderate: 100 mmHg < PaO_2_/FiO_2_ ≤ 200 mmHg, severe: PaO_2_/FiO_2_ ≤ 100 mmHg) [16].

### Statistical analysis

All categorical variables were presented as numbers with percentages, and all continuous variables were presented as medians with interquartile ranges (IQRs). In analyzing sepsis patients, we used the Mann–Whitney U test to compare continuous variables and the chi-square or Fisher’s exact test to compare categorical variables. Logistic regression analysis was performed using covariates that were significant (*P* < 0.05) on univariate analysis to identify risk factors for ARDS; a forward stepwise multivariate logistic regression model was used to screen independent predictors for ARDS. In analyzing patients who developed ARDS, we used the Kruskal–Wallis H test to compare continuous variables and the chi-square test for categorical variables. Ordered polytomous logistic regression was used to confirm the independent risk factors for the severity of ARDS. We used IBM SPSS for Windows version 24.0 for all statistical analyses.

## Results

### Baseline characteristics

**Fig. 1:**
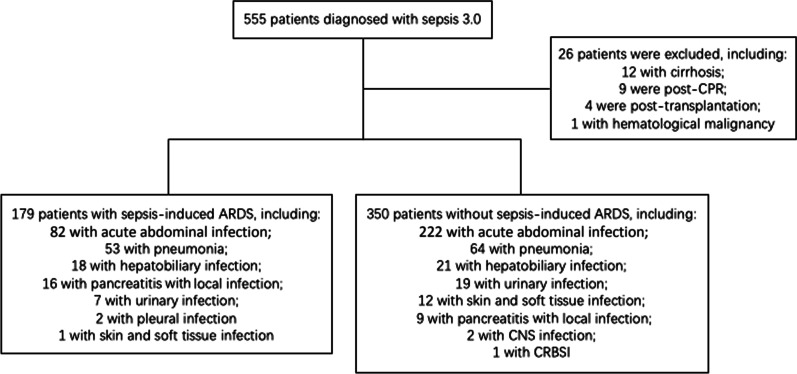
Flow chart of patient’s who were enrolled in this study. CPR, cardiopulmonary resuscitation; ARDS, acute respiratory distress syndrome; CNS, central nervous system; CRBSI, catheter related blood stream infection

**Fig. 2:**
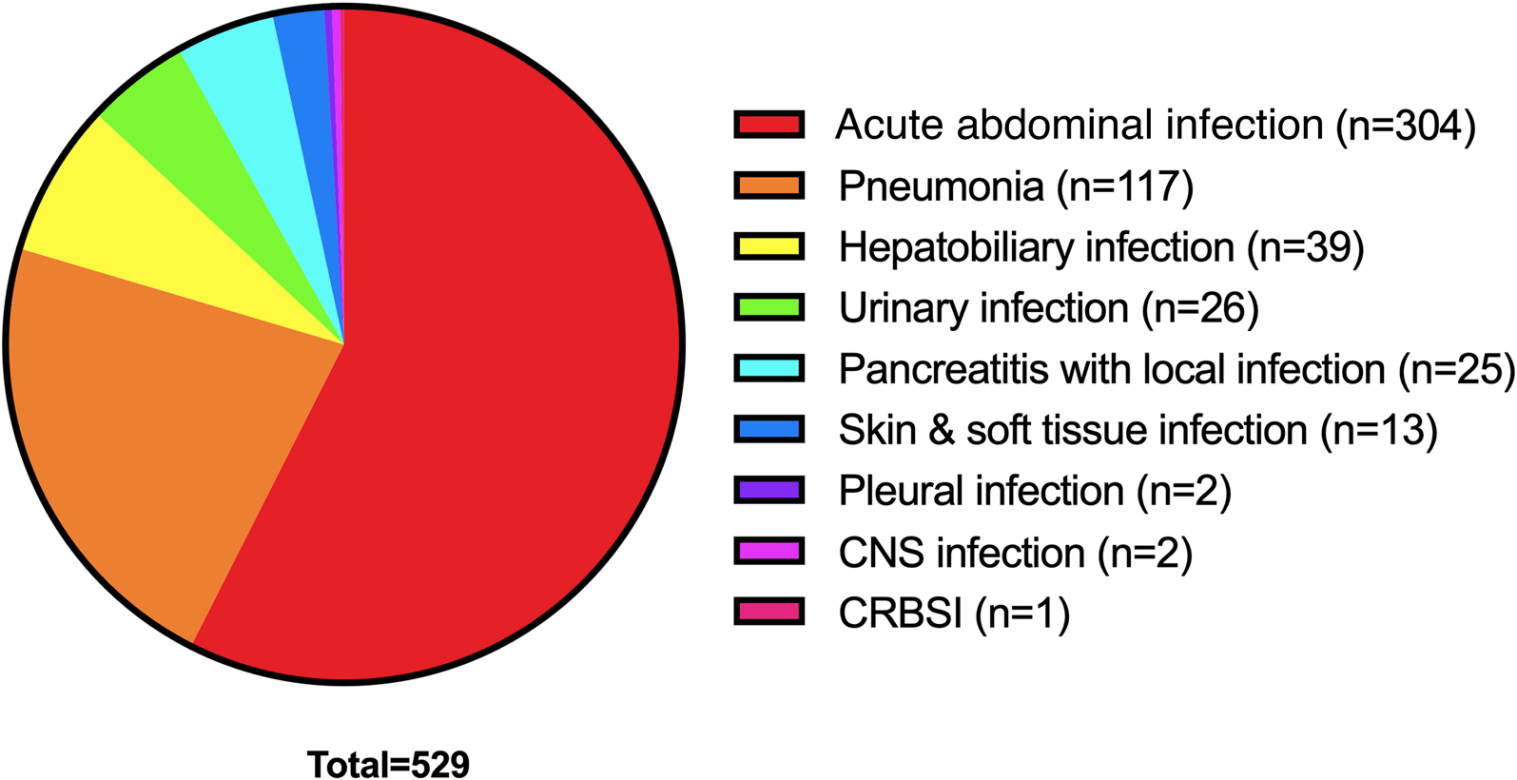
Distribution of infection site of sepsis patients. CNS, central nervous system; CRBSI, catheter related blood stream infection

A total of 555 patients who met the inclusion criteria were screened, and 529 patients were enrolled (Fig. 1). The most frequent source of sepsis sites were the acute abdominal infection (n = 304), followed by the pneumonia (n = 117) and hepatobiliary system infection (n = 39). The distribution of infection sites is demonstrated in Fig. 2. The incidence of septic shock was 46%. A total of 179 (34%) patients developed ARDS during ICU stay. The median age of ARDS patients was 66 (IQR 53–76), 134 (75%) were males. 6% had a history of drinking and 18% had a history of smoking. Additionally, 350 (66%) of the septic patients did not develop ARDS.

According to a comparison of the patients without ARDS to sepsis-induced ARDS patients, there were significant differences in the variables including age, sex, comorbid cancer, infection site (pneumonia, acute abdominal infection, pancreatitis with local infection, skin and soft tissue infection), emergency admission, septic shock, SOFA score, non-pulmonary SOFA score and APACHE II score (*P* < 0.05) (Table 1).

**Table 1:** Comparisons of baseline characteristics between ARDS and non-ARDS patients

Variable	ARDS (N = 179)	Non-ARDS (N = 350)	*P* value
Sex (male/female)	134 (75%)/45 (25%)	230 (66%)/120 (34%)	**0.019**
Age (years)	66 (53, 76)	70 (60, 78)	**0.003**
History of drinking	10 (6%)	27 (8%)	0.236
History of smoking	32 (18%)	50 (14%)	0.170
*Comorbidities*			
COPD	7 (4%)	17 (5%)	0.296
HTN	67 (37%)	120 (34%)	0.267
CHD	32 (18%)	79 (23%)	0.126
DM	37 (21%)	70 (20%)	0.470
Cancer	32 (18%)	90 (26%)	**0.026**
CKD	12 (7%)	25 (5%)	0.504
Hematological disorder	1 (1%)	2 (1%)	0.734
*Infection site*			
Pulmonary/Non-pulmonary	53 (30%)/126 (70%)	64 (18%)/286 (82%)	**0.002**
Pancreatitis with local infection	16 (9%)	9 (3%)	**0.002**
Acute abdominal infection	82 (46%)	222 (63%)	** < 0.001**
Hepatobiliary system	18 (10%)	21 (6%)	0.067
Urinary tract	7 (4%)	19 (5%)	0.296
Skin and soft tissue	1 (1%)	12 (3%)	**0.034**
Central nervous system	0 (0%)	2 (1%)	0.437
Pleural	2 (1%)	0 (0%)	0.114
CRBSI	0 (0%)	1 (0%)	0.663
Emergency admission	87 (49%)	213 (61%)	**0.004**
Septic shock	118 (66%)	125 (36%)	** < 0.001**
APACHE II	16 (13, 20)	14 (10, 18)	** < 0.001**
SOFA	9 (7, 11)	7 (4, 8)	** < 0.001**
Non-pulmonary SOFA	6 (4, 8)	5 (3, 6)	** < 0.001**

Values are presented as median (interquartile range) or number (%); Significant *P* values are in bold form

ARDS, acute respiratory distress syndrome; COPD, chronic obstructive pulmonary disease; HTN, hypertension; CHD, coronary heart disease; DM, Diabetes mellitus; CKD, chronic kidney disease; CRBSI, catheter related bloodstream infection; APACHE II, acute physiology and chronic health evaluation II; SOFA, sequential organ failure assessment

### Comparison of prognosis between ARDS and non-ARDS patients

The median length of mechanical ventilation was 114 (IQR 46–250) hours versus 34 (IQR 13–113) hours in ARDS patients and non-ARDS patients (*P* < 0.001), respectively. The median length of ICU stay was 7 (IQR 4–15) days versus 4 (IQR 2–8.25) days (*P* < 0.001). Moreover, both the ICU mortality rate (23% versus 10%) and 28-day mortality rate (47% versus 24%) were significantly higher in the ARDS group than in the non-ARDS group (*P* < 0.001). The analysis results of prognostic factors are listed in Table 2.

**Table 2:** Prognostic analysis between ARDS and non-ARDS patients

Variable	ARDS (N = 179)	Non-ARDS (N = 350)	P value
Length of mechanical ventilation (hours)	114 (45.5, 230)	34.2 (13, 113)	** < 0.001**
Length of ICU stay (days)	7 (4, 15)	4 (2, 8.25)	** < 0.001**
Length of ICU stay for survived patients (days)	8 (5, 15.5)	4 (2, 7.75)	** < 0.001**
ICU mortality rate (%)	23%	10%	** < 0.001**
28-day mortality rate (%)	47%	24%	** < 0.001**

Significant *P* values are in bold form. ARDS, acute respiratory distress syndrome; ICU, department of intensive care unit

### Risk factors for ARDS

A total of thirteen variables (Table 1) differed significantly between ARDS patients and non-ARDS patients. We took into account the clinical significance of the variables and finally screened out nine (sex, age, emergency admission, pneumonia, pancreatitis with local infection, septic shock, SOFA score, non-pulmonary SOFA score and APACHE II score) by univariate analysis (Table 3). Five variables remained significant after multivariate analysis (Table 4). Among them, pneumonia [odds ratio (OR), 3.486; 95% confidence interval (CI), 1.890—6.430, *P* < 0.001], pancreatitis with local infection (OR 3.601; 95% CI 1.429—9.073, *P* = 0.007), septic shock (OR 2.163; 95% CI 1.429—3.275,* P* < 0.001), SOFA score (OR 1.241; 95% CI 1.155—1.333,* P* < 0.001) and non-pulmonary SOFA score (OR 2.849; 95% CI 2.113—3.841,* P* < 0.001) were independent risk factors for ARDS in sepsis patients.

**Table 3:** Univariate analysis of risk factors for ARDS

Variable	OR (95% CI)	*P* value
Sex (male)	1.554 (1.038–2.326)	**0.032**
Age (years)	0.990 (0.984–0.997)	**0.003**
Emergency admission	0.676 (0.420–0.869)	**0.004**
Pancreatitis with local infection	3.719 (1.609–8.595)	**0.002**
Pneumonia	1.880 (1.235–2.861)	**0.002**
Septic shock	3.482 (2.385–5.084)	**< 0.001**
APACHE II	1.029 (1.016–1.042)	**< 0.001**
SOFA	1.150 (1.115–1.186)	**< 0.001**
Non-pulmonary SOFA	1.092 (1.059–1.125)	**< 0.001**

Significant *P* values are in bold form. ARDS, acute respiratory distress syndrome; OR odds ratio; CI, confidence interval; APACHE II, acute physiology and chronic health evaluation II; SOFA, sequential organ failure assessment

**Table 4:** Multivariate analysis of risk factors for ARDS

Variable	OR (95% CI)	P value
Pneumonia	3.486 (1.890–6.430)	**< 0.001**
Pancreatitis with local infection	3.601 (1.429–9.073)	**0.007**
Septic shock	2.163 (1.429–3.275)	**< 0.001**
SOFA	1.241 (1.155–1.333)	**< 0.001**
Non-pulmonary SOFA	2.849 (2.113–3.841)	**< 0.001**

Significant *P* values are in bold form. ARDS, acute respiratory distress syndrome; OR odds ratio; CI, confidence interval; SOFA, sequential organ failure assessment

### Risk factors for the severity of ARDS

**Fig. 3:**
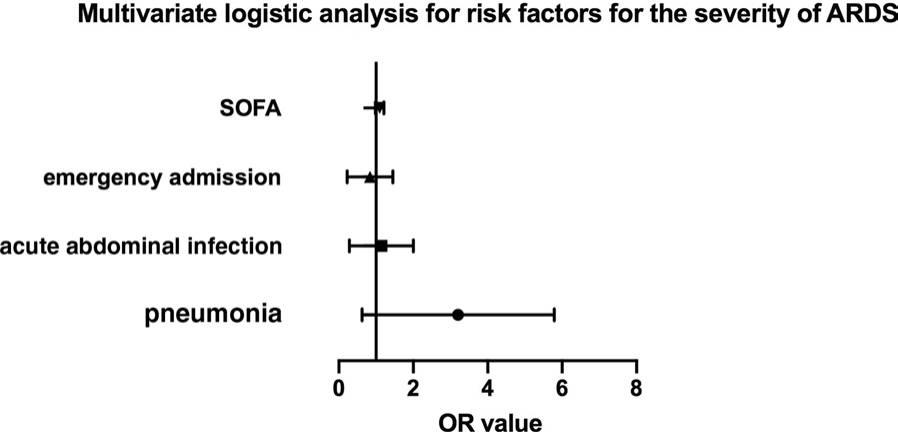
Forest plot for risk factors of the severity of ARDS. Pneumonia remains the only significant risk factor for increased severity of ARDS in patients with sepsis (OR 2.512, 95% CI 1.039–6.067, p = 0.041). Other risk factors enrolled in the analysis include emergency admission (OR 0.685, 95% CI 0.311–1.513, p = 0.349), acute abdominal infection (OR 0.926, 95% CI 0.411–2.088, p = 0.852), SOFA (OR 1.093, 95% CI 0.985–1.212, p = 0.093). ARDS, acute respiratory distress syndrome; OR, odds ratio; CI, confidence interval; SOFA, sequential organ failure assessment

Among the 179 ARDS patients, 39 (22%) were diagnosed with mild ARDS, 109 (61%) were diagnosed with moderate ARDS and 31 (17%) were diagnosed with severe ARDS. Variables that showed significant differences between ARDS and non-ARDS patients were included in the comparison among the three groups with different severities of ARDS. As a result, patients with pneumonia (mild ARDS vs moderate ARDS vs severe ARDS, 15% vs 24% vs 55%, *P* < 0.001) and higher SOFA score (7.92 vs 9.35 vs 9.58, *P* = 0.014) were more likely to develop severe ARDS. On the contrary, acute abdominal infection was the main cause of mild and moderate ARDS compared to severe ARDS (62% vs 51% vs 26%, *P* = 0.010). Prognostic factors showed significant differences in 28-day mortality rate (mild ARDS vs moderate ARDS vs severe ARDS, 25.6% vs 42.2% vs 41.9%, *P* = 0.023) and 90-day mortality rate (30.8% vs 43.1% vs 61.3%, *P* = 0.038) among the three groups (Table 5). Ordinal multivariate logistic regression revealed that patients with pneumonia (OR 2.512; 95% CI 1.039–6.067; *P* = 0.041) had significant correlation with increased severity of ARDS (Fig. 3).

**Table 5:** Comparisons between patients with mild, moderate, and severe ARDS

Variable	Mild (N = 39)	Moderate (N = 109)	Severe (N = 31)	*P value*
Sex (male/female)	31 (79%)/8 (21%)	80 (73%)/29 (27%)	23 (74%)/8 (26%)	0.781
Age (years)	66 (52, 77.5)	66 (54, 77)	62 (53.5, 72)	0.538
History of drinking	6 (15%)	22 (20.2%)	4 (12.9%)	0.595
History of smoking	2 (5%)	8 (7%)	0 (0%)	0.274
*Infection site*				
Pulmonary/Non-pulmonary	6 (15%)/33 (85%)	26 (24%)/83 (76%)	17 (55%) /14 (45%)	** < 0.001**
Pancreatitis with local infection	5 (13%)	11 (10%)	2 (7%)	0.684
Acute abdominal infection	24 (62%)	55 (51%)	8 (26%)	**0.010**
Emergency admission	26 (67%)	52 (48%)	9 (29%)	**0.007**
Septic shock	24 (62%)	72 (66%)	22 (77%)	0.720
APACHE II	16 (12, 19.5)	16 (12, 20)	16 (14, 21.5)	0.283
SOFA	8 (6, 10)	9 (8, 11)	10 (8, 12)	**0.014**
Non-pulmonary SOFA	5 (4, 7)	6 (5, 7.25)	6 (4.5, 8)	0.251
Length of mechanical ventilation (hours)	144 (49, 235)	112 (44, 224)	106 (48, 236)	0.861
Length of ICU stay (days)	8 (3, 13)	7 (4, 15)	6 (3, 16.5)	0.772
Length of hospital stay (days)	19 (12, 28)	22 (13, 38)	24 (6, 31)	0.356
ICU mortality rate (%)	17.9%	25.7%	22.6%	0.616
28-day mortality rate (%)	25.6%	42.2%	41.9%	**0.023**
90-day mortality rate (%)	30.8%	43.1%	61.3%	**0.038**

Significant *P* values are in bold form. ARDS, acute respiratory distress syndrome; COPD, chronic obstructive pulmonary disease; HTN, hypertension; CHD, coronary heart disease; DM, Diabetes mellitus; CKD, chronic kidney disease; CRBSI, catheter related bloodstream infection; APACHE II, acute physiology and chronic health evaluation II; SOFA, sequential organ failure assessment; ICU, department of intensive care unit

### Prognosis of pulmonary ARDS

We further analyzed the prognostic profile of ARDS patients between pneumonia and non-pulmonary infection. Significant differences were found in length of mechanical ventilation (170 h vs 105.5 h, *P* = 0.013), length of ICU stay (11 days vs 7 days, *P* = 0.007), ICU mortality rate (39.6% vs 16.7%, *P* = 0.001), 28-day mortality rate (62.3% vs 33.3%, *P* < 0.001) and 90-day mortality rate (64.2% vs 34.9%, *P* < 0.001) (Table 6).

**Table 6:** Comparison between pulmonary and non-pulmonary groups in sepsis‐associated ARDS

Variable	Pulmonary ARDS (n = 53)	Non-pulmonary ARDS (n = 126)	*P value*
Sex(female/male)	14 (26.4%)/39 (73.6%)	31 (24.6%)/95 (75.4%)	0.799
Age (years)	70 (55, 80)	64 (53, 72.75)	**0.017**
History of smoking	14 (26.4%)	18 (14.3%)	0.130
Septic shock	39 (73.6%)	79 (62.7%)	0.161
APACHE II	17 (13, 22)	15.5 (12, 20)	0.063
SOFA	10 (7, 12)	9 (7.25, 11)	0.158
PaO_2_/FiO_2_	126.0 (90, 167.5)	171.525 (137.26, 202.25)	** < 0.001**
Length of mechanical ventilation (hours)	170 (46, 375)	105.5 (40.25, 203.5)	**0.013**
Length of ICU stay (days)	11 (4, 27)	7 (4, 12)	**0.007**
Length of hospital stay (days)	27 (12, 40)	20 (12, 32)	0.082
ICU mortality rate (%)	21 (39.6%)	21 (16.7%)	**0.001**
Hospital mortality rate (%)	21 (39.6%)	22 (17.5%)	**0.002**
28-day mortality rate (%)	33 (62.3%)	42 (33.3%)	** < 0.001**
90-day mortality rate (%)	34 (64.2%)	44 (34.9%)	** < 0.001**

Significant *P* values are in bold form. ARDS, acute respiratory distress syndrome; APACHE II, acute physiology and chronic health evaluation II; SOFA, sequential organ failure assessment; ICU, department of intensive care unit

## Discussion

In this study, we reported a rate of 34% of ARDS incidence in sepsis patients diagnosed according to sepsis 3.0 criteria. We also compared the clinical characteristics between patients with or without ARDS. We determined five risk factors for ARDS in sepsis patients. Furthermore, we figured out for the first time that pneumonia is significantly associated with an increased severity of ARDS. Our study adds additional evidence about sepsis patients who are at high risk of developing ARDS.

The 34% incidence of ARDS was the highest in the literature to date [10–14]. Possible reasons include: (1) the increased severity of sepsis in our cohort, as 46% patients developed septic shock, which was also a higher rate than that in previous studies [17]; (2) the improved clinical recognition of ARDS at our center. The 28-day mortality rate of ARDS patients from our cohort was 47%, which is similar to the 28-day mortality rate of sepsis-induced ARDS patients in a previous study (42%) [13], reflecting that our data is consistent and convincible.

Our study found a younger median age in patients with sepsis-induced ARDS (66 years vs. 70 years, *P* < 0.05). We noticed that the results of 3 studies, including ours, showed that sepsis patients who developed ARDS were younger than those who did not (*P* < 0.05), although age was not an independent factor for developing ARDS [10, 11]. ARDS is a clinical syndrome characterized by severe inflammatory responses accompanied by immune activation [18]**.** A set of functional and structural changes in the immune system, such as a decreased response to vaccination and inflammation response, are thought to be crucial components of aging [19]. This decreased response might be the underlying reason why the median age of ARDS patients is lower than that of non-ARDS patients. Further studies may focus on this interesting clinical phenomenon in critically ill patients.

Our study reported a significantly worse prognosis of patients with sepsis-induced ARDS compared with those without ARDS, regarding to the length of mechanical ventilation, length of ICU stay, and 28-day mortality, which is consistent with the data in the literature. Researchers reported that sepsis-induced ARDS leads to prolonged recovery from lung injury and delayed extubation [20, 21]. Moreover, the progression to ARDS is associated with an increased risk of in-hospital death in sepsis patients [22, 23]. Therefore, it is urgent to determine the risk factors of sepsis patients who developed ARDS.

We confirmed that septic shock is an independent risk factor for ARDS, which is consistent with previous studies [10, 11]. Septic shock is a severe form of sepsis in which the underlying circulatory, cellular, and metabolic abnormalities are enough to substantially increase the risk of death. Septic shock has long been recognized as a trigger factor for acute lung injury (ALI) and ARDS [14]. Iscimen et al. reported that 44% of septic shock patients developed ALI in their ICU [14], which was basically consistent with the rate in our ICU (48.6%). Patients with septic shock should undergo a more precise respiratory function assessment.

Moreover, we revealed a strong relationship between the infection site and the development of ARDS in sepsis patients, including pneumonia and pancreatitis with local infection. It has been reported that pneumonia is the most common source of infection in ARDS patients and also a risk factor for ARDS [24, 25]. In this case, ARDS may be caused by both direct lung injury and indirect systemic inflammatory response of sepsis. In patients with pancreatitis, ARDS is thought to be caused by severe systemic inflammatory response that leads to increased permeability of the endothelial and epithelial barriers, with leakage of protein-rich exudates into the alveolar space and interstitial tissues, thereby affecting oxygenation and gas exchange [26–28].

We also confirmed that SOFA and non-pulmonary SOFA scores were significantly associated with an increased risk of ARDS. Apart from SOFA, other assessment scores including lung injury prediction score (LIPS)[29], early acute lung injury (EALI) score[30], and APACHE II score have been reported to be associated with the development of ARDS. Studies of patients in the emergency department have shown that APACHE II score is an independent risk factor for ARDS in sepsis patients [10, 22], while SOFA score plays an independent role in bacteremia patients in ICU [12] which is inconsistent with our result. This may be explained by the fact that most patients admitted to the emergency department are at an early stage of the disease process and have not yet developed organ failure, while ICU patients have already developed complex organ dysfunction. This may remind clinicians to pay more attention to respiratory function in patients with other organ dysfunction, such as acute kidney injury or coagulation dysfunction.

Furthermore, we confirmed an association between pneumonia and increased severity of ARDS. Moreover, patients with pneumonia-induced ARDS showed significantly worse PaO_2_/FiO_2_ index, longer duration of mechanical ventilation and higher mortality rate. In the case of pneumonia, the pulmonary defense system can trigger an immune response to microbes, resulting in profound local and systemic inflammatory responses that may develop into ARDS, or severe ARDS [31, 32]. Nam et al. reported a significantly higher rate of pneumonia patients among 28-day non-survivors with ARDS caused by bacteremia-induced sepsis in Korea [11]. However, other studies showed no relationship between pneumonia and increased mortality [24, 33, 34]. Indeed, these results may not be directly comparable due to different inclusion criteria. In addition, this may be due to the differences in populations between the studies, such as Asian and non-Asian. We hypothesize that beneficial measurements and interventions should be implemented more aggressively in pneumonia patients to reduce the progression to severe ARDS, which may enhance the prognosis of ARDS caused by pulmonary sepsis.

ARDS is a clinical syndrome with a high rate of under-diagnosis. Up to 40% of ARDS patients cannot be clinically recognized quickly enough [1]. A better understanding of the risk factors for ARDS in sepsis patients will improve the ability of clinicians to identify ARDS as early as possible. Moreover, several studies have shifted the emphasis from clinical risk factors to biomarkers for development of ARDS, which is also a strategy for better precision medicine for ARDS. [35–37] Larger studies should be carried out to provide appropriate interventions for specific patients.

The limitations of this study are as follows: 1. Our study is a retrospective study, and the homogeneity of the data cannot be guaranteed, which may affect the results of the study. 2. The severity of the disease in the selected population is high, which cannot represent the clinical characteristics of all patients with sepsis–induced ARDS. 3. This study is a single-center study, and the results cannot be generalized to all patients with sepsis-induced ARDS or all the patients with severe ARDS.

## Conclusion

Our study in the northeastern China showed that pneumonia, pancreatitis with local infection, septic shock, SOFA score and non-pulmonary SOFA score were independent risk factors for the development of ARDS in sepsis patients. In addition, pneumonia was significantly related to increased severity of ARDS and increased mortality. Multicenter, prospective studies are needed to better understand this severe clinical syndrome with high heterogeneity.

## Data Availability

The data could be shared if readers contact the authors and explain why they would like to access the data and material. Corresponding author should be contacted if someone wants to request the data from this study.
